# Metabolic Alterations in *Pisum sativum* Roots during Plant Growth and Arbuscular Mycorrhiza Development

**DOI:** 10.3390/plants10061033

**Published:** 2021-05-21

**Authors:** Oksana Shtark, Roman Puzanskiy, Galina Avdeeva, Vladislav Yemelyanov, Alexey Shavarda, Daria Romanyuk, Marina Kliukova, Anastasia Kirpichnikova, Igor Tikhonovich, Vladimir Zhukov, Maria Shishova

**Affiliations:** 1 Department of Biotechnology, All-Russia Research Institute for Agricultural Microbiology, Pushkin, 196608 St. Petersburg, Russia; daria-rom@yandex.ru (D.R.); marina.kliukova@gmail.com (M.K.); igor.tikhonovich49@mail.ru (I.T.); vzhukov@arriam.ru (V.Z.); 2 Laboratory of Analytical Phytochemistry, Komarov Botanical Institute of the Russian Academy of Sciences, 193022 St. Petersburg, Russia; puzansky@yandex.ru (R.P.); stachyopsis@gmail.com (A.S.); 3 Faculty of Biology, St. Petersburg State University, 199034 St. Petersburg, Russia; avdeevag21@yandex.ru (G.A.); bootika@mail.ru (V.Y.); nastin1972@mail.ru (A.K.); 4 Center for Molecular and Cell Technologies, St. Petersburg State University, 199034 St. Petersburg, Russia

**Keywords:** *Pisum sativum*, arbuscular mycorrhiza, plant growth and development, root, metabolic profile

## Abstract

Intensive exchange of nutrients is a crucial part of the complex interaction between a host plant and fungi within arbuscular mycorrhizal (AM) symbiosis. For the first time, the present study demonstrates how inoculation with AMF *Rhizophagus irregularis* affects the pea (*Pisum sativum* L.) root metabolism at key stages of plant development. These correspond to days 21 (vegetation), 42 (flowering initiation), and 56 (fruiting-green pod). Metabolome profiling was carried out by means of a state-of-the-art GC-MS technique. The content shifts revealed include lipophilic compounds, sugars, carboxylates, and amino acids. The metabolic alterations were principally dependent on the stage of plant development but were also affected by the development of AM fungi, a fact which highlights interaction between symbiotic partners. The comparison of the present data with the results of leaf metabolome profiling earlier obtained did not reveal common signatures of metabolic response to mycorrhization in leaves and roots. We supposed that the feedback for the development and symbiotic interaction on the part of the supraorganismic system (root + AM fungi) was the cause of the difference between the metabolic profile shift in leaf and root cells that our examination revealed. New investigations are required to expand our knowledge of metabolome plasticity of the whole organism and/or system of organisms, and such results might be put to use for the intensification of sustainable agriculture.

## 1. Introduction

Arbuscular mycorrhiza (AM) is a widespread symbiosis in nature formed by the vast majority of land plants with arbuscular-mycorrhizal fungi (AMF) of the Glomerymycota division [1]. AMF are obligate biotrophs: their life cycle is impossible without the supply of sugars and lipids from the host plant [2,3,4]. In plants, AM also performs several functions even though most plants can grow and develop without AM fungus in favorable conditions of mineral and water supply. AM thus improves plant water and mineral nutrition, including the uptake of inorganic phosphate (Pi), under natural conditions of strong competition for soil nutrients [1,5,6]. Likewise AM symbioses improve plant tolerance to both abiotic and biotic stresses [7,8,9]. Plant mycorrhizal dependency varies owing to the plant species/genotype, the type of fungus, the ecological adaptation of partners, and agroclimatic and soil conditions [1,6,10,11,12]. Therefore, in order to effectively apply plant symbiosis with AMF in sustainable agriculture, it is necessary to study in detail the ecological, genetic, physiological, and biochemical aspects of this interaction.

Recently, a paradigm has been shifted from low-throughput, single end-point bioassays to systemic biology approaches. Thus, researchers have been employing so-called ‘omic’ tools, including transcriptomics, proteomics, and metabolomics [13,14,15,16]. Metabolomics is a particularly powerful tool for discovering how a plant’s physiological/biochemical status varies under different environmental conditions [13,14]. Numerous metabolomic studies revealed mycorrhiza-mediated changes in the biochemical composition of leaf tissues [11,17]. Some evidence for AM-induced root metabolome changes were shown for barrel medick (*Medicago truncatula*) [18], tomato [19,20], wheat [21], and soybean [22,23]. It is now known that the establishment of symbiosis with AMF affects the primary metabolism of the plant, thereby facilitating the exchange of photosynthates with AMF. The carbon supply leads to the transport of a significant amount of sugars to roots, as well as organic acids due to intensification of the Krebs cycle. Reprogramming secondary metabolite biosynthesis in mycorrhizal roots results in an increase in plant resistance to biotic and abiotic stresses. In addition, activation of the AMF phenylpropanoid pathway causes an increase in the diversity of secondary metabolites, a phenomenon which might serve to improve the quality of plant foods and pharmaceutical raw materials [24,25]. Especially noted was that the shifts in the plant metabolome triggered by symbiosis depend on both plant and AMF species. Therefore, an expansion of our knowledge of the metabolic changes within the interactions between different plants and AMF could allow for a more efficient use of this symbiotic association to increase plant productivity [25].

The pea (*Pisum sativum* L.), an important legume crop, is able to form symbiosis with beneficial soil microorganisms, including AMF. Some pea genotypes exhibit relatively high mycorrhiza-dependence [26]. However, most of the studies conducted under model conditions [5,10,27,28,29,30], and in the field [31], indicate that the pea has a low responsiveness to mono-inoculation with AMF. Often, a noticeable positive effect of mycorrhization on the growth parameters of pea plants is observed only when they are grown under stressful conditions [27,29]. However, mycorrhization causes physiological and biochemical changes in the aboveground organs of pea plants [5,29]. In particular, Shtark et al. [5] showed that mycorrhization led to the retardation of plant development, which was also associated with an extended vegetation period and with an increase in the seed biomass of inoculated plants. Furthermore, during flowering and fruiting, the leaf metabolic profiles of inoculated pea plants shifted towards the profiles of the uninoculated plants at earlier developmental stages. A similar trend has been described for the seed proteomic profile in the pea line that had earlier been selected as highly sensitive to double inoculation with both AMF and nodule bacteria [26,32]. The line responded to inoculation by prolongation of seed maturation, manifested by up-regulation of the synthesis of proteins involved in cellular respiration, protein biosynthesis, and down-regulation of late-embryogenesis abundant protein synthesis. In contrast, the pea line with low responsivity to combined inoculation demonstrated lower levels of the proteins related to cell metabolism [32]. Thus, the analysis of the effect of inoculation with soil microorganisms on the physiology and biochemistry of pea plants, including the use of metabolomic profiling, can be useful for sustainable agriculture in the search for optimal technologies for the cultivation of different pea cultivars. At the same time, data about the effect of AM symbiosis on the pea metabolome are limited to a few studies focused only on changes in aboveground organs, such as leaves [5,29,33] and seeds [34]. There are no data on changes in the metabolome of pea roots under the influence of AMF.

The aim of the present study was to analyze the effect of inoculation with AMF *Rhizophagus irregularis* on the root metabolome of pea plants at the key stages of plant development with application of gas chromatography-mass spectrometry (GC-MS). This paper presents the results of a comparative analysis of changes in the metabolite profiles of roots of non-mycorrhizal and mycorrhizal pea plants. Previously, AM-induced changes in the metabolite profiles of plant leaves from the same vegetation experiment were published in Shtark et al. [5]. Despite the fact that plants did not show a strong growth response to the inoculation, and their photosynthetic activity was not affected by mycorrhization, that study revealed significant metabolic alterations occurring in pea leaves during the AM development [5]. The present investigation showed that the pea root metabolome is very sensitive to AMF inoculation and depends on the stage of AM development. A comparison of AM-induced metabolic changes in the roots and leaves did not reveal common trends of metabolic response to mycorrhization.

## 2. Results

### 2.1. Arbuscular Mycorrhiza Development and Root Growth

**Figure 1:**
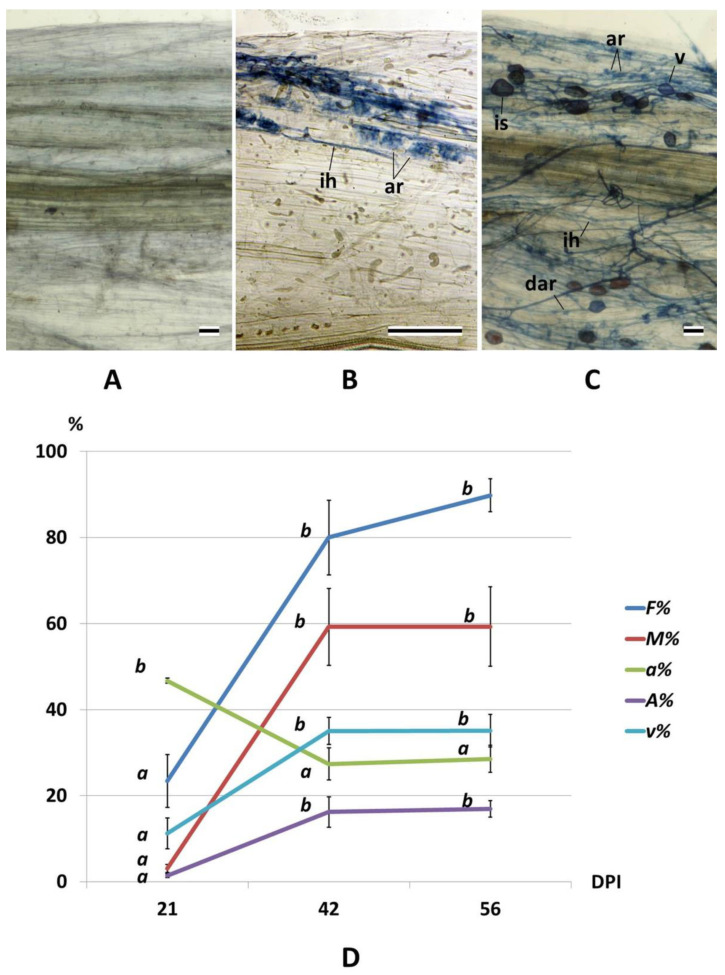
Arbuscular mycorrhiza (AM) development by *R. irregularis* in cleared pea cv. Finale secondary roots stained with Sheaffer Black Ink. (**A**) Root fragment of a non-inoculated plant. (**B**,**C**) Intraradical mycelium patterns most characteristic of various stages of AM development. (**B**) The stage of intensive functioning of arbuscules and the exchange of metabolites between the fungus and the plant (most typical for the time point 21 DPI in this experiment). (**C**) The stage of formation of storage and reproductive organs (vesicles and spores) (most typical for the time point 42 and 56 DPI in this experiment). *ih*, intercellular hypha; *ar*, arbuscule; *dar*, degrading arbuscule; *v*, vesicle; and *is*, intraradical spore. Scale bar, 50 μm. (**D**) Quantitative characteristics of AM development in the inoculated plants: *F%* is the frequency of mycorrhizal colonization, *M%* is the intensity of intraradical mycelium development, *a%* is the arbuscule abundance in mycorrhizal root fragments, *A%* is the saturation of the root system with arbuscules, and *v%* is the vesicle and spore abundance in mycorrhizal root fragments. The values for each parameter, which are not significantly different from each other (*p* ≤ 0.05), are marked with the same letter. Error bars represent standard errors.

Mycorrhizal fungus structures were not found in the roots of non-inoculated cv. Finale pea plants (Figure 1a), which indicated that the growth substrate was well sterilized and there was no cross-contamination of the plants during vegetation. In the plants inoculated with *R. irregularis*, the pattern of intraradical mycelium growth differed significantly at the time point of 21 days post inoculation (DPI) from that of the time points of 42 and 56 DPI (Figure 1b,c). The frequency of mycorrhizal colonization (*F%*; Figure 1d) and the intensity of intraradical mycelium growth (*M%*; Figure 1d) reached relatively high values by 42–56 DPI, whereas at 21 DPI it was very low. At the same time the abuscule abundance (*a%*; Figure 1d) was significantly higher at 21 DPI than that at 42 and 56 DPI. The dynamics of the change in the saturation of the root system with arbuscules (*A%*, Figure 1d) was similar to that for *F%* and *M%*. The vesicle and spore abundance (*v%*; Figure 1d) was higher at 42–56 DPI in comparison with that at 21 DPI. This indicates that, at 21 DPI, mycorrhizal infection was just beginning to spread, while mycorrhiza had a high functional activity; at 42–56 DPI mycorrhiza was well developed and the fungus was in a state of storing nutrients and reproducing. Nodules were not found in the root system of the analyzed plants.

Despite the fact that AM colonization was abundant in the plant roots, no effect of mycorrhiza on the root fresh weight was found (data not shown). No effect on the shoot fresh weight was also found in the studied pea genotype under these experimental conditions [5].

### 2.2. Changes in the Metabolite Profiles of Pea Roots

#### 2.2.1. General Characteristics of the Metabolite Profile

The metabolite profiles of pea roots included 253 metabolites (Table S1), of which 88 were identified completely and another 39 were identified up to their class (pentoses, hexoses, sterols, etc.). Twenty two amino acids (16 proteinogenic), about two dozen carboxylic acids, energy metabolism intermediates, 19 fatty acids and their derivatives, as well as nitrogenous bases, sugar alcohols, sterols, and others were detected. In the obtained profiles, sugars (about 50), including pentoses, hexoses, and oligosaccharides, were the most widely represented metabolites.

#### 2.2.2. Identification of Differences in the Metabolite Profiles of Pea Roots during Plant Development

**Figure 2:**
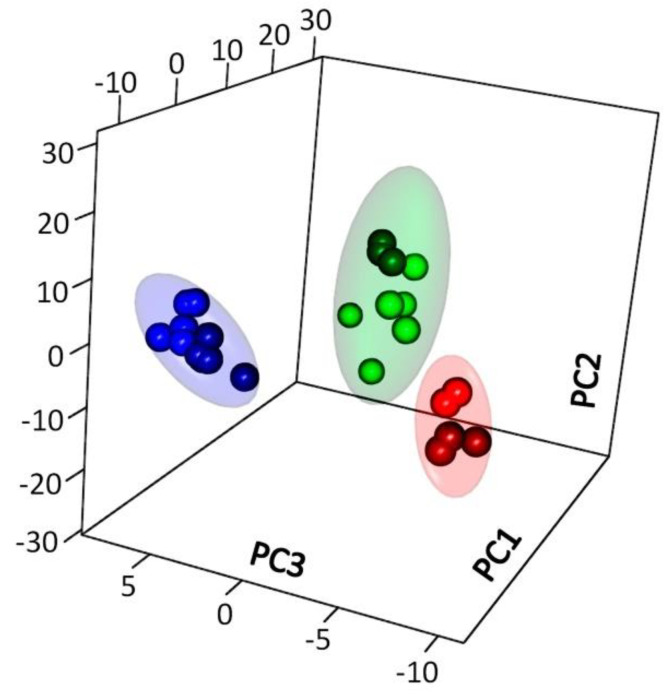
PCA score plot from principal component analysis of metabolite profiles of pea cv. Finale roots sampled at days 21 (in red), 42 (in green), 56 (in blue) infected with AM fungus *R. irregularis* (darker dots) or not (lighter dots). Ellipses are 90% CI.

In order to identify and visualize the similarities and differences of pea root metabolomes, they were presented in lower-dimensional spaces as obtained with two unsupervised methods. First, the principal component method was used (PCA, Figure 2 and Figure S1a). It can be seen that the resulting profiles are grouped according to the age of the plant. The metabolite profiles are represented in the space of PC1 and PC3 (Figure S1a). All time points are different in the PC3 space, explaining 11.9% of the variance, and the “42nd day” point is different in the PC1 space, representing 35.7%. The tendency of grouping in accordance to mycorrhization also occurs and relates to PC2 (18.4%).

Application of the metabolite content as a means of accounting for proximity is not always the best form of measurement, since proximity may vary on account of the normalization method (by fresh weight, protein, etc.). Therefore, the metabolite content correlation was considered as a measure of proximity of metabolite profiles and then the dimension was reduced using MDS. In Figure S1b, the metabolite profiles are presented in the space of the first and third dimensions, the picture is similar to the one obtained using PCA.

**Figure 3:**
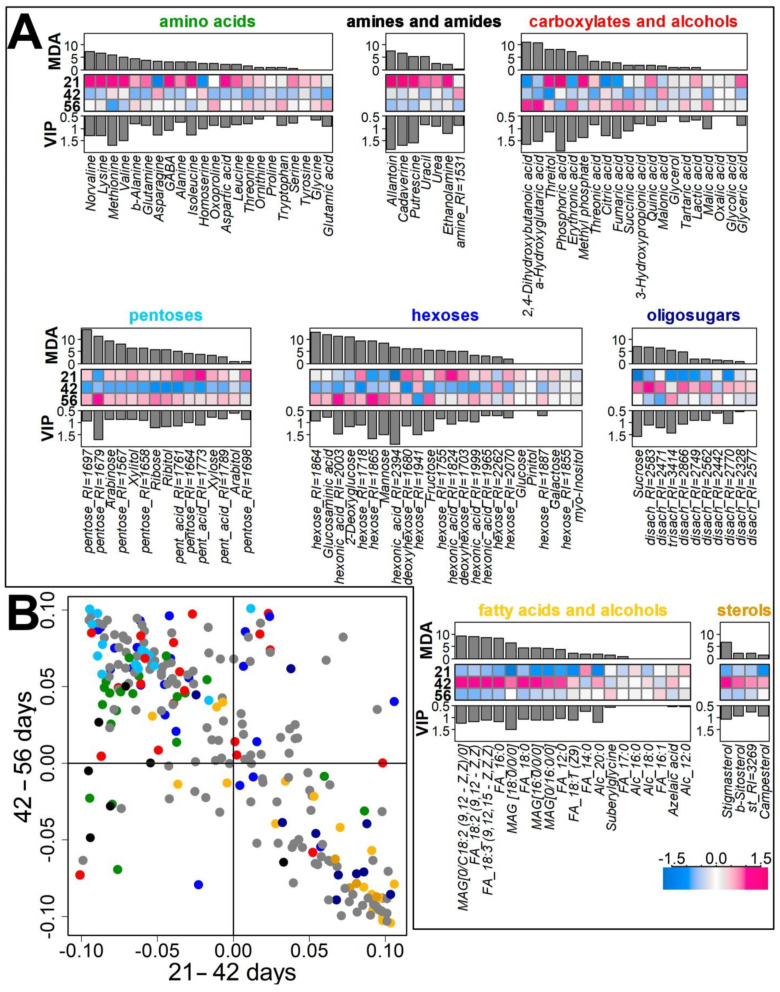
Analysis of age dependence of metabolite profile diversity. (**A**) Heatmaps of arbitrary normalized metabolite levels in pea cv. Finale roots during development of control plants sampled at days 21, 42, and 56. The top barplots are Mean Decrease Accuracy (MDA) obtained by the Random Forest, the bottom barplots are VIPs from PLS-DA. (**B**) SUS-plot: metabolite scattering in the space of loadings from OPLS-DA models for comparing roots of 21 and 42 day old plants (abscissa) and 42 and 56 day old (ordinate); positive values correspond to higher level at a later stage. Colors mirror chemical classes. Grey circles correspond to unidentified metabolites. “MAG”—monoacylglycerol; “FA”—fatty acid; “Alc”—fatty alcohol; “st”—sterol; RI—Kovatc retention index.

The subsequent analysis of profile diversity as a variable of age was carried out using the supervised methods of PLS-DA and Random Forest (Figure 3). The resulting PLS-DA model contained 2 predictive components (*t*), explaining 35 and 12% of the variance, respectively, at R^2^Y = 0.926, Q^2^Y = 0.907. It should be noted that *t1* was associated with differences in the metabolite profiles of 42-day-old plants, and *t2* with the one in plants of all ages, especially plants at 21 and 56 days old.

Figure 3A shows heatmaps of average values (logarithmic and normalized) combined with barplots of VIP values and Mean Decrease Accuracy (MDA) of Random Forest. These indicate how strongly the content of metabolites is related to the age of the plant.

A number of amino acids (primarily norvaline, valine, lysine, methionine) and amines (allantoin, cadaverine, putrescine) were characterized by high VIP and MDA values and maximum content at the beginning of plant development.

As can be seen, at the age of 42 days, plants were characterized by the increased content of a number of sugars. A particularly strong relationship was established for sucrose. A similar pattern of content was typical for most other disaccharides. For many monosaccharides, the opposite dynamics was revealed with a minimum content at day 42, for example, for such major sugars as ribose, arabinose, and fructose.

The highest content of lipophilic compounds was also observed at this period. These included fatty acids (especially unsaturated ones) and sterols.

For a more detailed analysis of metabolite profile dynamics at each time interval (21d–42d and 42d–56d), two OPLS-DA models were constructed. In both cases, the predictive component was associated with about 33% of the variance at the 21–42 day stage and 34% at the 42–56 day stage (Q^2^ > 0.9). Thus, the scale of changes at these stages was similar. The analysis of factor loadings (Figure 3B and Figure S2) showed that their values were in a strong inverse relationship (Spearmans’s correlation coefficient *r_s_* = −0.66, *p* < 10^−15^). This pattern was observed in both control and mycorrhizal plants (Figure S2).

### 2.3. Identification of Differences in the Metabolite Profiles of Pea Roots during Mycorrhization

#### 2.3.1. Vegetation Stage (21 Days)

**Figure 4:**
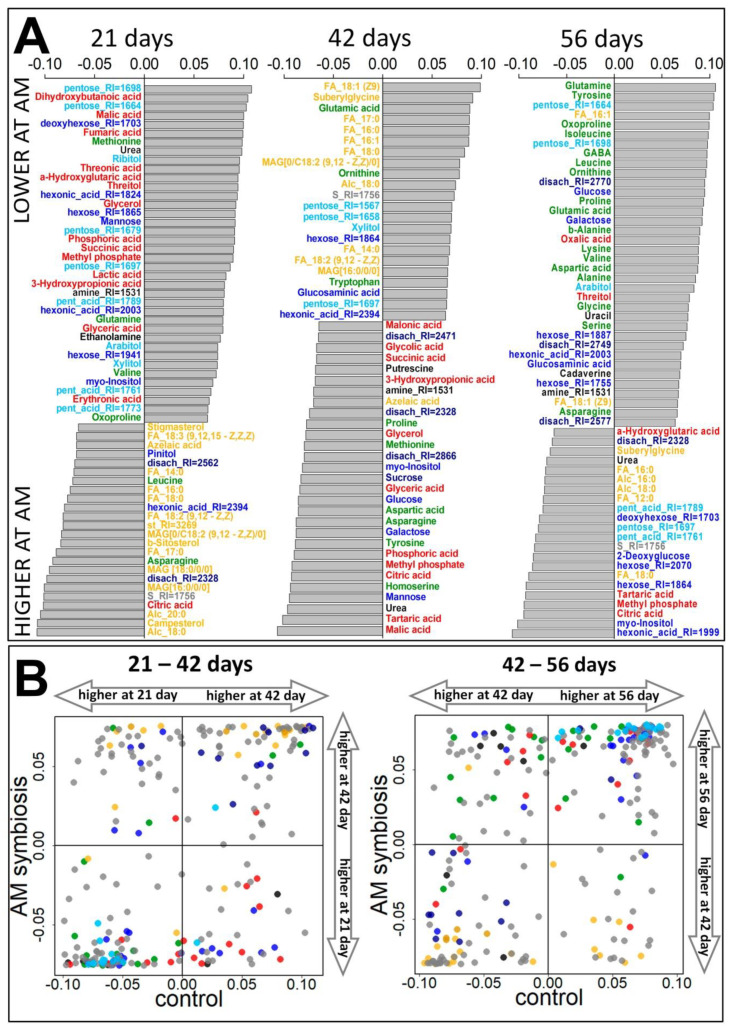
Influence of mycorrhiza on the metabolome of pea cv. Finale roots sampled at 21, 42, and 56 day. (**A**) Barplots of the loadings of predictive components (VIP > 1) from OPLS-DA models for comparison of control and mycorrhized roots, positive values correspond to a higher content during mycorrhization. (**B**) Influence of mycorrhiza on the metabolome dynamics. Metabolites scattering in the space of the loadings from OPLS-DA models for comparing adjacent time points under control (abscissa) and mycorrhization (ordinate). Color code is the same as in Figure 3.

The OPLS-DA classification showed that 34% (R^2^Y = 0.99, Q^2^Y = 0.84) of the variance of the metabolite content was associated with the predictive component. Thus, during this period, the role of mycorrhization in the formation of the root metabolite profile was significant. Figure 4 shows the loading diagram of the predictive component with VIP values >1. Positive values correspond to a higher content in the presence of the AM fungus. It can be seen that the roots of mycorrhizal plants were dominated by carboxylates and monosaccharides. In contrast, lipophilic compounds (sterols, fatty acids, acylglycerols, terpenes) were characterized by a decrease in their level during mycorrhization. For amino acids, the opposite trends of change were identified. Thus, in the presence of the fungus, the content of methionine, glutamine, valine, and oxoproline increased, while asparagine and leucine decreased.

**Figure 5:**
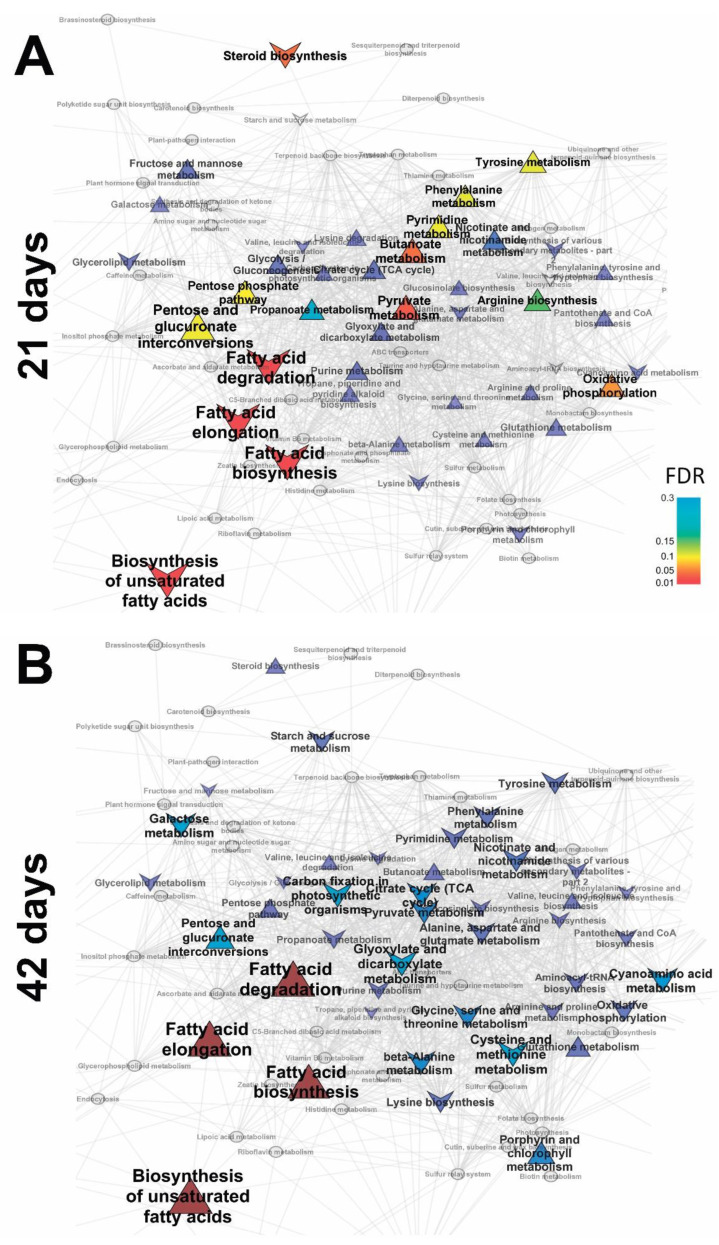

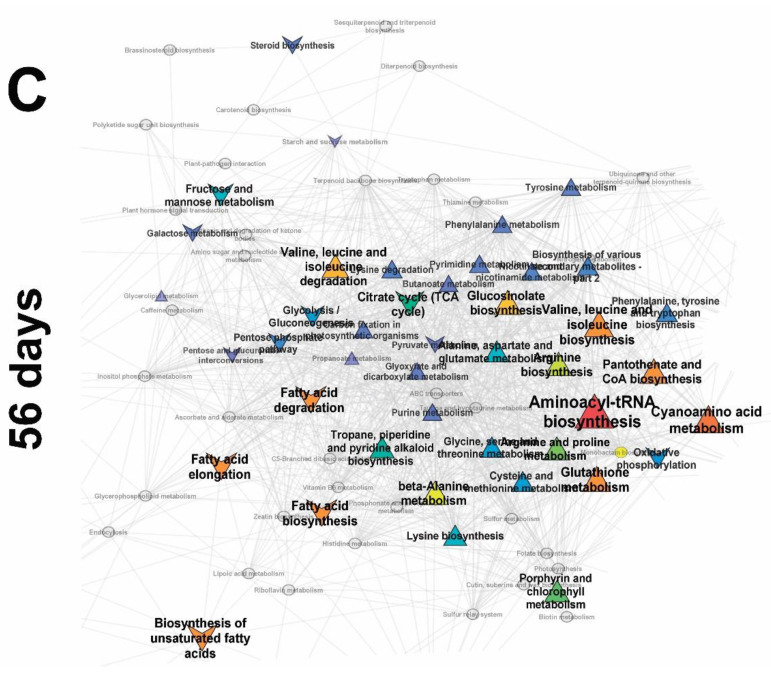
Enrichment analysis. Graph of the KEGG pathways. Nodes are pathways, if paths have common metabolites, then they are connected by edge, attracting nodes with strength proportional to number common metabolites. Color denotes significance of influence of mycorrhization on the path (FDR), derived from MSEA predictive component loadings of OPLS-DA models for comparison of the metabolome of the mycorrhized and control pea cv. Finale roots sampled at 21 (**A**), 42 (**B**) and 56 day (**C**). Size denotes strength of influence (|NES|), upward direction denotes activation with mycorrhiza, downward denotes repression.

To determine which biochemical pathways were affected by mycorrhization, a Metabolite Set Enrichment Analysis (MSEA) was performed based on the values of the predictive component loadings and the KEGG pathway metabolite sets. As presented in Figure 5, the intensity of the pathways of fatty acid synthesis and sterols was decreased at day 21. At the same time, a specific increase in the content of intermediates of oxidative phosphorylation, carboxylate exchange, and pentoses was revealed, which indicates the activation of these pathways. With caution, we can talk about aromatic amino acids exchange increase.

#### 2.3.2. Flowering Stage (42 Days)

According to the OPLS-DA model, at this stage, 30% (R^2^Y = 0.98, Q^2^Y = 0.92) of the metabolites content variance was associated with the predictive component, which is similar to that of day 21. Figure 4A shows the loading diagram of the predictive component with VIP values >1. Positive values correspond to a higher content in plants in the presence of an arbuscular fungus. In the roots of mycorrhizal plants, lipophilic compounds, fatty acids, and acylglycerols dominated. As shown by the enrichment analysis (Figure 5), the main metabolite effect of mycorrhization at this stage was focused on the activation of the fatty acid metabolism, which is significantly different from the observations at the 21st day.

Additionally, in the roots of mycorrhizal plants, a higher content of certain amino acids was found, for example, in tryptophan, glutamate, and ornithine, whereas content of homoserine, tyrosine, asparagine, aspartate, methionine, proline, as well as amines and urea, was higher in the control plants. Remarkably, among the metabolites with a high content in non-mycorrhizal plants, a large number of carboxylates were noted, including those associated with various energy cycles, for example, malate, citrate, glycolate, succinate, malonate, etc. In control plants, a higher content of a number of sugars, such as glucose, mannose and sucrose, was observed.

#### 2.3.3. Frooting (Green Pod, 56 Days)

The model constructed using OPLS-DA showed that 33% of the variance in the content of metabolites (R^2^Y = 0.99, Q^2^Y = 0.94) was associated with a predictive component similar to the previous stages. The analysis of the loadings of the predictive component (Figure 4A) showed that positive values corresponding to a higher content under mycorrhizal conditions are inherent for many amino acids. Bidirectional changes in the content of fatty acids were observed depending on mycorrhizal infection. Saturated fatty acids and alcohols (12:0, 16:0, 18:0) had a higher content in the roots of control plants, and monounsaturated (18:1, 16:1) in mycorrhizal plants. It was noted that, at day 56, enrichment analysis showed a decrease in the pathways associated with the exchange of fatty acids in the presence of a symbiont (Figure 5). The differences in the sugar content were also multidirectional: major sugars such as glucose, galactose, and a number of unidentified monosaccharides (and their derivatives) were found in greater quantities in mycorrhizal plants. Control plants were characterized by higher content of a number of sugar acids and deoxyhexoses. In the absence of a symbiotic fungus, the root metabolome was characterized by a high content of carboxylates: citrate and tartrate.

#### 2.3.4. Influence of Mycorrhiza on Plant Development

Taking into account the fact that differences in metabolite profiles at each stage are the result of metabolic changes that take place over the course of development, we undertook a comparison of metabolomes at the two examined stages of 21–42 and 42–56 days in the control and during the development of symbiosis. The loadings of the obtained OPLS-DA models are shown in Figure 4B and Figure S3. Positive loading values correspond to an increase in the content at the next stage. In both cases, a positive correlation was observed (*r_s_* ≈ 0.55, *p* << 0.001). The results indicate that metabolite changes during the development were similar with mycorrhization and without a symbiotic fungus. The number of metabolites that deviate from this dependence (signed in Figure S3) at the 21–42 day stage include carboxylates, as well as some lipophilic compounds and sugars. At the 42–56 day stage, the group of different metabolites consisted of amino acids, carboxylates, and lipophilic compounds. The analysis of the models obtained showed that the differences in dynamics are well reflected in the differences of content at the corresponding time points.

### 2.4. Correlation Analysis of the Metabolite Pools

**Figure 6:**
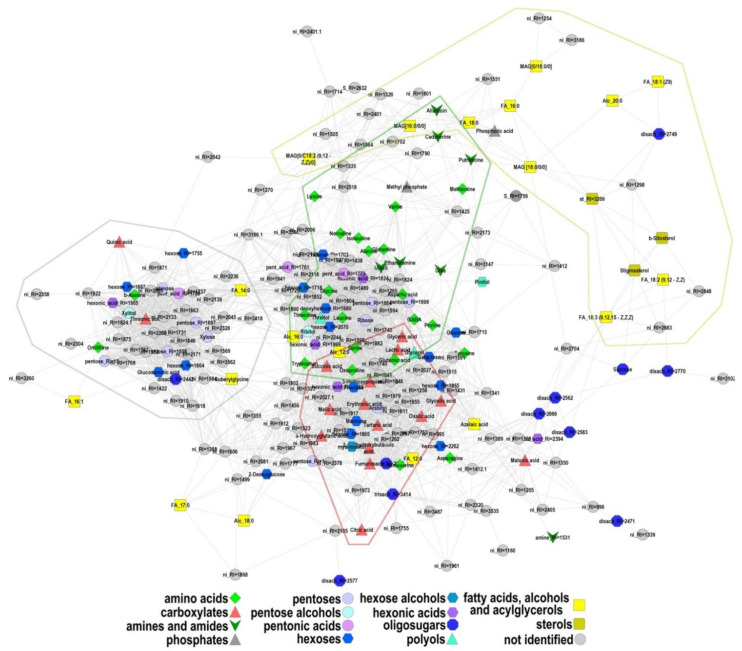
Mapping of metabolites by correlation of levels. Nodes correspond to metabolites, the shape and color reflect the chemical nature of the compound. Edges correspond to the strong Pearson correlations (r > 0.9) of the normalized average content in the variant (age-mycorrhization). Lines bound areas of related metabolites.

To resolve systemic metabolic relations which are relevant to development and mycorrhization, we analyzed the correlations of the average metabolite levels in each experimental variant. An interesting feature of the distribution of the Pearson correlation coefficient is the high frequency of high positive values (Figure S4). This may indicate that physiological changes are highly coordinated with changes in metabolite levels over the course of development. In order to visualize the structure of links between the metabolite pools, metabolites were mapped by strong correlations (|r| > 0.9, *p* < 0.01). Positive correlations bring nodes together, negative and zero correlations push them apart. The resulting graph is shown in Figure 6. At the center of the graph, the area of carboxylates can be distinguished; adjacent to it is the amino acid and amines segment, and lipophilic compounds are located on the periphery. The weak correlation of lipophilic compounds is in good agreement with the relatively weak association (total metabolites) of their metabolic pathways with the rest of the metabolism. This is noticeable in the KEGG metabolite pathway maps (Figure 5). Thus, correlations of changes in the metabolite content during physiological changes can be defined as much by chemical compound similarities (areas of similar compounds) as by metabolite affinities (pathway connections) that are also cross-defined.

#### Comparison of the Effect of Mycorrhization on the Leaf and Root Metabolome

**Figure 7:**
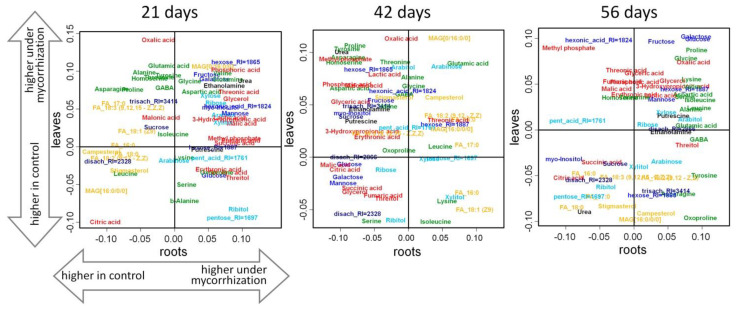
Comparison of the effect of mycorrhization on the roots and leaves of pea cv. Finale roots sampled at days 21, 42, and 56. SUS plots: scattering in the space of factor loadings of predictive components from OPLS-DA models for control and mycorrhized plant classification (abscissa—roots, ordinate—leaf), positive loadings correspond to higher content under mycorrhization. Color code is the same as in Figure 3.

To determine the specificity of the effect of mycorrhization on the metabolite profiles of roots and to identify possible differences from those previously shown for leaves [5], we compared the factor loadings of predictive components from the OPLS-DA models for the classification of mycorrhizal and control plants (Figure 7). It emerged that there was no significant correlation in the case of plants at days 21 and 42. The data obtained indicate the absence of a general response to mycorrhization in roots and leaves. Only at the 56th day did the weak reliable correlation occur (*r_s_* = 0,29, *p* = 0.02), an indication of the appearance of similarities in the effect of mycorrhization on the organs under analysis.

Such a radical difference in the effect of mycorrhization in leaves and roots may be due to several factors, including metabolite differences among the respective organs (Figure S5a). In order to determine this, we compared differences in mycorrhization effects depending on the organ expressed as the difference between loadings of the predictive components (p_roots_-p_leaf_) from the OPLS-DA models for classification of control. Mycorrhized plants and the general dissimilarity of leaf and roots were then expressed as loadings of predictive components from OPLS-DA models for roots and leaf classification (Figure S5b). Interestingly, at the first two stages, the differences in the mycorrhizal colonization effect were significantly negatively associated with the difference between leaves and roots in general (for 21 days: *r_s_* = −0.41, *p* < 0.01; for 42 days: *r_s_* = −0.28, *p* = 0.02). It can be concluded that the metabolite response of the organ to mycorrhization depends on its metabolite characteristics. At days 21 and 42, the metabolites that had a tendency to accumulate more in the roots in comparison to the leaves (abscissa) tended to have their accumulation in the roots stimulated by mycorrhization to a lesser extent than in the leaves (Figure S5b). This was especially typical for many amino acids, the content of which was much higher in roots. At the same time, the increase in their content at day 21 and 42 under the effect of mycorrhization was noted only for a small number of them (Figure 4). At the same stage, the level of many amino acids in the leaves increased markedly under the influence of mycorrhizal colonization. A similar situation was observed with carboxylates at day 42. This may suggest that mycorrhization is associated with high levels of amino acids (and some other metabolites in the lower right sector of Figure S5b) in plants. However, it may be the case that growth of their synthesis occurs in leaves under the influence of mycorrhization—in the roots it was high from the very beginning, and thus did not require such a strong induction. At day 56 day, the level of amino acids in the roots, as well as in the leaves, increased (Figure 7). This may be a result of the depletion of the amino acid pool, which decreased dramatically between days 21 and 42 (Figure 3) in both control and mycorrhizal plants (Figure 4 and Figure S2).

## 3. Discussion

Plants have developed different adaptations to cope with unfavorable environmental conditions. Nutrient deficiency, particularly concerning soil-immobile elements such as P, usually trigger two events: changes in root architecture and establishment of arbuscular mycorrhizal (AM) symbiosis [35,36]. According to the literature, mycorrhization might result in the accumulation of proteins, carbohydrates, primary and secondary metabolites, probably owing to a better supply of phosphate and nitrogen [37,38,39,40]. Nonetheless, this accumulation does not always result in an increase of fresh weight. The low growth response of *P. sativum* to AMF mono-inoculation was shown in our previous research and some other publications [5,28,29,30]. Precise analysis of pea plant development during AM symbiosis revealed a very important event, namely that the pea plants inoculated with AMF prolonged the active phase of the vegetation period [5]. The effect was clearly distinguished with the application of GC-MS profiling of pea leaves. This investigation aimed to use the same experimental setup (host plant, AM inoculation, conditions of growing, etc.) to discover possible alterations in root metabolome under direct interaction with AMF.

The pea root metabolomes obtained with GC-MS were less complex than those in leaves. A similar effect was earlier defined for other species grown under different environmental conditions [41]. Less diversity of carbohydrate metabolism in the roots is responsible for the lack of photosynthesis and associated carbohydrate metabolism pathways. Besides, limitation in monosaccharides in non-mycorrhizal pea plants might be linked to intensive exudation of primary metabolites into the rhizosphere. It involves different mechanisms, including passive losses and active exudation, but the exact way to regulate it is still poorly understood [42]. Plants are supposed to spend up to 20 to 40% of their photosynthetically fixed C in root exudates [43]. It is mainly suggested that exudates modify nutrient accessibility directly and through microbiota attraction. Some metabolites also might fulfill a regulatory role in plant development. For young plants, sugars efflux was shown to stimulate root elongation under P deficiency [44]. This might be the case for our data; under P-limitation at 21st DPI sugars were presumably transported out of the root system to facilitate root growth. At later stages of pea plant development, this effect disappeared.

A large number of carboxylates (for example, malate, citrate, glycolate, succinate, malonate, etc.) were noted among the metabolites with a high content in young non-mycorrhizal plants (Figure 2). These compounds are associated with various energy cycles that are in high demand at early stages of development. Carboxylates, especially citrate and malate, are also known to be secreted by the root system to facilitate nutrient absorption, including P, from poor soils [35]. Higher concentrations of different amino acids and amines most likely reflect intensive synthetic processes [23]. The latter are scarcely a required energy source and thus result in decrement to the levels of lipophilic compounds and sugars.

Our previously obtained data highlighted a strong dependence on the part of leaf metabolite spectrum on plant development stages [5]. In this investigation, the same tendency was easily estimated with a simple PCA for root metabolomes (Figure 2). The overwhelming majority of metabolites demonstrated non-monotonous alterations in pea root metabolic profiling. Sugars and lipophilic compounds were discovered to be the most variable. Most of the oligosaccharides, including sucrose, accumulated at 42 DPI, which was characterized with a transition to flowering (Figure 3). Similar alterations were detected for lipophilic compounds like free fatty acids, sterols, etc. Preparation for flowering is a fundamental stage of plant development. The priority of different organs within the organism is supposed to be intensively revised and new cross-interactions and a sink/source relationship between plant organs have been established. The next stage tested, fruiting (green pod, 56 DPI), also caused alterations in root metabolome. Mycorrhization caused elevation of amino acid levels as well as the level of monounsaturated (18:1, 16:1) fatty acids.

Along with metabolic rearrangement during pea plant development, a possible AM contribution was expected (Figure 4). Pea roots were intensively sensitive to AMF inoculation. Mycorrhization at a vegetative stage resulted in higher accumulation of carboxylates and monosaccharides, while representation of lipophilic compounds (fatty acids, acylglycerols, sterols and other terpenes) were decreased. Multidirectional sets of changes were revealed for amino acids. Over transit to flowering, AM pea roots differed in the elevation of lipophilic compounds level and the expanding diversity of amino acids. At the beginning of fruiting, the inoculated pea roots still accumulated amino acids as well as monounsaturated fatty acid.

Further enrichment analysis (Figure 5) revealed successive changes of metabolic pathways from the turnover of carboxylates, pentoses, and intermediates of oxidative phosphorylation (21 DPI) to activation of fatty acid metabolism in mycorrhizal pea roots (42 DPI). At 56 DPI, enrichment analysis showed a decrease in the pathways associated with the exchange of fatty acids in the presence of the symbiont and intensification of amino acid and carbohydrates metabolism.

The difficulty of evaluating the exact mycorrhization effect on the root metabolite profile is related to a complex interaction between plant and fungi. Actually, these biochemical alterations are the sum of such an interaction in the symbiotic pair. It has to be admitted that the intensive changes of AMF structures from penetrated hyphae to arbuscules, mycelium and vesicles are the result of different metabolic and physiological activities. Thus, the influence of AMF on metabolism in the examined profiles is quite possible.

Furthermore, increased amounts of asparagine and aspartic acid in arbuscule-containing cells are associated with higher nitrogen availability. Arginine is shown to be desaminated in the intraradical mycelium and nitrogen is then transferred to the host plant in a form of ammonium [45]. The extent of the amino acid balance in inoculated roots is known to be very dependent on P supply [23]. We observed a weak elevation of the aromatic amino acid metabolism at early stages of vegetative growth. These are known to be precursors of phenolic compounds. AM induces stimulation of phenolic compound synthesis in roots that are known to positively regulate AMF spore germination, hyphal growth, and the colonization process [23]. AMF could be interfering with the sugar balance. The hexoses assimilated by AMF can further be transformed into trehalose and glycogen in the intraradical mycelium of AMF [46]. Recent investigations demonstrate that the AM fungus also obtains fatty acids from the host plant. They stimulated FA synthesis, transformed and deposited it as triacylglycerols, and transported it through mycelia to support AMF metabolism, growth, and sporulation [4,47,48].

It should be noted that metabolites establish clusters according to their chemical properties. The mapping of these metabolites was done according to strong correlations (|r| > 0.9, *p* < 0.01) with average levels of metabolites content in each experiment (Figure 6). In the center of the obtained graph, the carboxylate single compact group can be distinguished with its attached amino acid and amine segment. Despite the importance of sugar levels and their alteration at different points of time, we did not find a single region for these metabolites. Weak connectivity of fatty acids with other metabolites could be explained on the basis of some metabolic apartness of lipid biosynthesis. This is clearly illustrated in Figure 5, where pathways are mapped by the presence of common metabolites in metabolome. Weak connectivity between fatty acids and derivates is more intriguing, because they are closely metabolically linked. Just as for carbohydrates as for fatty acids, wide spreading on the graph may mirror the involvement of these metabolites in different biochemical processes with different functions. It is determined by more complicated quantitative regulation during processes of development and mycorrhization.

The final step of our investigation involved comparing AM-induced metabolic changes in the roots (the model of this investigation) and leaves (the model of the previous one, [5]). For that we used the same time stages of pea plant development (21, 42, and 56 DPI). The PLS-DA models developed, and further comparison of differences between loadings of the predictive components, did not reveal a common schedule of metabolic response to mycorrhization in root and leaf (Figure 7). To explain this fact we can only make the suggestion that the metabolic specificity between leaf and root is highly sensitive to the mycorrhizal symbiosis effect. Moreover, this phenomenon is a very complex, multicomponent reciprocal response which includes regulation of plant metabolism itself during development and demands of AMF. We know too little to assess the exact relationship between those processes yet. Differences in response to mycorrhization between roots and leaves are related to their metabolic distinctions. In particular, metabolites with a tendency to accumulate more in roots tend to be less stimulated by mycorrhiza (Figure S5b). From this we assumed that compounds supporting the growth of mycorrhizal fungus (especially amino acids) accumulate in the roots. At the same time, after the initiation of mycorrhiza growth, continuous consumption of these compounds is observed. This, in turn, strongly activates their synthesis in leaves, which is reflected in the rise of the level of such compounds.

## 4. Materials and Methods

### 4.1. Biological Material, Experimental Conditions, and Collection of Plant Material

Plants of the pea (*Pisum sativum* L.) cv. Finale (Cebeco, Rotterdam, The Netherlands; [49]) were grown in 300-mL ceramic flower pots with sterile soil and quartz sand mixture (1:2 *v/v*), supplemented with 1g·L^−1^ Ca_3_PO_4_ as a source of insoluble phosphate. A loamy sandy soddy-podzolic soil obtained from Gatchinsky district, Leningrad Oblast, Russia, with the following characteristics was used: pH (KCl) 4.8; 3.6% organic matter; 35 mg·kg^−1^ available K_2_O (extraction with 0.2 N HCl); 33 mg·kg^−1^ available P_2_O_5_ (extraction with 0.2 N HCl); 28,7 mg-equivalent.kg^−1^ hydrolytic acidity; 98 mg-equivalent.kg^−1^ base exchange materials. CaCO_3_ (1.44 g·kg^−1^) was added to the soil to correct pH. Pots with the soil-sand mixture were autoclaved twice with a two-day interval for 60 min at 134 °C and 0.22 MPa to remove soil microbiota and kept for a month to eliminate volatile toxic compounds. Half of the pots were supplemented with 15 g·L^−1^ *Rhizophagus irregularis* BEG144 (the International Bank for the Glomeromycota, Dijon, France) inoculum before planting; the other half was left as control. To produce the inoculum of the fungus for this experiment, mycorrhizal *Sorghum* sp. plants were grown in pot cultures (see Shtark et al. [5] for details). Pea seeds were surface-disinfected as follows: 1 min in 96% ethanol, a rinse with sterile water, 8 min in a 5% NaClO aqueous solution, and a thorough rinse with sterile water. Disinfected pea seeds were germinated on sterile humid vermiculite in Petri dishes for 3 days at 27 °C in the dark. Two pea seedlings of equal size were planted into each pot. Plants were grown in a constant environment chamber (model VB 1514, Vötsch, Germany) at 16/8 h and 24/22 °C day/night regime, 75% relative humidity, and around 10,000 lux illumination. The pot cultures were fertilized once a week with 0.5× Hoagland’s solution [50] without phosphate (0.15 L·L^−1^ of the growth substrate), and watered as needed. Thus, pea plants were grown under conditions of a deficiency of available phosphate.

The plants were analyzed at three time points corresponding to specific developmental stages for the growth of this species: (i) 21 days post inoculation (DPI) (vegetative stage, first leaf with two pairs of leaflets, complex tendril); (ii) 42 DPI (reproductive stage, first open flower); and (iii) 56 DPI (reproductive stage, pod fill, green seeds fill the pod cavity). The developmental stages were selected in accordance to Knott [51]. Ten or more plants per treatment were taken at random at each stage and root fresh biomass and other plant growth parameters (see Shtark et al. [5] for details) were estimated. For AM analysis, fragments of lateral roots were collected individually from each plant (*n* = 10) in 2 mL Eppendorf™ tubes and were stored at –20 °C. For metabolome analysis, randomly selected root samples from 3 to 5 plants were allocated to a single biological replicate, weighed and snap-frozen in liquid nitrogen in 2 mL Eppendorf™ Safe-Lock tube, and then stored at −80 °C. Root metabolome analysis was performed in three biological and two technical replicates.

### 4.2. Analysis of AM Development

Root samples were cleared with 10% KOH and stained with Sheaffer Black Ink according to Vierheilig et al. [52]. After staining the roots were washed once with distilled water and covered in glycerol; root fragments totaling a length of 30 cm for each plant were laid out on a glass slide, covered with a second slide, and squashed. The AM development was examined using a light microscope (Axiovert 35, Zeiss, Jena, Germany). Visual observations were documented using an EOS10D camera (Canon, Tokyo, Japan), with the help of DSLR Remote for Windows Version 2.0 (Breeze Systems Limited, Camberley, UK), and processed by Adobe Photoshop for Windows Version CC (Adobe Systems Inc., San Jose, CA, USA). The AM development was quantitatively assessed according to Trouvelot et al. [53] by the following parameters: *F%* = frequency of mycorrhizal colonization (reflects the proportion of mycorrhized root fragments among all those analyzed), *M%* = intensity of intraradical mycelium development (reflects the proportion of the root length colonized by the fungus), *a%* = arbuscule abundance in mycorrhizal root fragments (characterizes the functional state of the fungus), *A%* = saturation of the root system with arbuscules (*A% = M × a/100*), and *v%* = vesicle and spore abundance in mycorrhizal root fragments (characterizes the transition of the fungus to the stage of storing nutrients and reproduction). For statistical analysis, the parameters were subjected to arcsine transformation to normalize data [54]. Data on mycorrhiza development was processed with one-way ANOVA with normal distribution. The SPSS 12.0 package (SPSS Inc. Chicago, IL, USA) was used for ANOVA. All data were expressed as mean ± standard error. The differences were considered as significant at the confidence level of *p* ≤ 0.05.

### 4.3. Sample Preparation for Metabolome Analysis

Plant material for the analysis of the root metabolome was collected in the same experiment as that earlier used for pea leaf metabolome analysis [5]. The samples of plant material (0.2–0.3 g) were ground as described by Puzanskiy et al. [55] and subjected to a single-stage extraction with 2 mL methanol: chloroform: water (5:2:1) mixture. Tissue debris was removed by centrifugation at 12,000× *g* for 10 min at −5 °C. The supernatant was collected and evaporated in a vacuum evaporator (Eppendorf, Germany). The dried material was dissolved in pyridine with the internal tricosane standard (*n*C23, tricosane). The samples were then supplied with the sylilating agent BSTFA:TMCS 99:1 (Sigma-Aldrich) and derivatizated at 90 °C for 20 min.

### 4.4. GC-MS Analysis

GC-MS analysis was performed with an Agilent 5860 chromatograph (Agilent Technologies, Santa Clara, CA, USA). Separation was provided on a DB-5MS capillary column 30 m long 0.25 mm in diameter, stationary phase film (95% dimethylpolyoxane, 5% diphenyl), thickness 0.25 μm. Helium flow rate was 1 mL/min. Inlet temperature was 250 °C at splitless mode. The temperature conditions of the column thermostat were the following: initial temperature of 70 °C, increased by 5 °C/min up to 320 °C. The peaks were recorded by an Agilent 5975S mass selective detector (Agilent Technologies, Santa Clara, CA, USA). Electron impact ionization was performed at 70 V and an ion source temperature of 230 °C.

### 4.5. GC-MS Data Interpretation

The analysis of the GC-MS data was processed using the PARADISe program (Department of Food Science Faculty of Science, University of Copenhagen, Denmark, [56]) in association with NIST MS Search (National Institute of Standards and Technology (NIST), Gaithersburg, MD, USA). In addition, we used the AMDIS (Automated Mass Spectral Deconvolution and Identification System, NIST, USA). The following mass-spectrometer libraries were used: NIST2010, GMD (the Golm Metabolome Database, Potsdam, Germany), MoNA (Massbank of North America, Davis, CA, USA), and in-house libraries of the Resource Center “The Development of Molecular and Cell Technologies” of the St. Petersburg University and Botanical Research Institute RAS (St. Petersburg, Russia). Retention indices (RI) were determined by calibration with standard alkanes.

### 4.6. Mathematical Analysis of Metabolome Data

Data was processed in the environment of the R language 3.6.3 “Holding the Windsock” (R Core Team, [57]). Data were normalized by the internal standard (nC_23_) and per mass. Outlying values were excluded on the basis of Dixon’s test. When metabolite detection was negative in one sample but positive in other replicated samples, it was postulated as technical error and missing values was imputed. Missing data imputation was performed by KNN (k-nearest neighbors) with *impute* R package [58]. Data was normalized per sample median, and log2-transformed and autoscaled. PCA (Principal Component Analysis, PCA) was realized with *pcaMethods* [59]. 3D graphics were performed with *rgl* package [60]. (O)PLS-DA made with *roopls* [61]. To test effects of class imbalance on classification, additional models were constructed after weighted centering or after class balancing through resampling. All significant effects on classification accuracy or feature selection (loadings and VIPs) were to be detected. Random Forest (RF) was held by *random Forest.* Class weighting was used to avoid class imbalance effects [62].

For enrichment analysis, the *fgsea* package was used [63]. As a ranking statistics factor, loadings of the predictive components from OPLS-DA models were used. Pathways associated with our metabolite set were extracted from KEGG with the *KEGGREST* package using *Pisum sativum* as reference organism [64]. The resulting lists of metabolites for pathways were manually curated and metabolites identified just up to class were included in the pathways related to these groups. Pathways were mapped in the Cytoscape software environment [65]. In the graph, nodes were assigned to KEGG pathways, and edges represent the presence of common metabolites.

Metabolites were mapped by significant (*p* < 0.05) and strong Pearson’s correlation coefficients (|r| > 0.9) of their arbitrary content within the Cytoscape. software environment [65], using “perfuse force directed layout”. A heatmap was made with the *ComplexHeatmap* package [66].

## 5. Conclusions

The data of our long-lasting investigation represent root metabolome alterations during host plant development. It has been clearly determined that transfer from vegetative growth, through flowering to fruiting, caused significant changes in the metabolic pathways that necessarily provided the required nutrients. The most intensive deviations occurred for sugars, lipophilic compounds, carboxylates, and amino acids. These data testified to the flexibility of metabolic networks specific to different stages of development. Besides that, rather intensive rearrangements were triggered by AM fungus interaction with pea roots. The appearance of different mycorrhizal structures enriches root metabolome profiles most probably due to the AM fungi metabolism and rearrangements of supply from pea leaves. Thus, root profile shifts result from a systemic metabolic response on the part of both host plant and symbiotic fungi. The most significant of these responses was related to lipophilic compounds, sugars, and amino acids. We suspect that the observed difference between the metabolic profile shift in leaf and root cells was caused by the feedback directed toward the development and symbiotic interaction of the supraorganismic system (root + AM fungi). Additional complicity of data analysis is also related to the well-known tissue specificity of metabolic pathways in different plant organs. The most important finding in our investigation is the estimation of the metabolic network adjustment for continuous developmental changes in pea plant roots hosting arbuscular mycorrhizal fungi. Both symbionts pass through physiological transformation and coordinate one another’s metabolism under unfavorable limitations in P supply. We have made only the first step in understanding the order of biochemical interactions and elucidating the signaling role of metabolites for mycorrhiza symbiosis. New investigations are required to expand our knowledge on metabolome plasticity of the whole organism and/or systems of organisms.

## Data Availability

Data is contained within the article or Supplementary Material.

## Supplementary Materials

The following are available online at https://www.mdpi.com/article/10.3390/plants10061033/s1, Figures S1–S5, Table S1 and raw chromatograms.
