# Supplementary material for: Metabolic Alterations in Pisum sativum Roots during Plant Growth and Arbuscular Mycorrhiza Development
Source: Plants (Basel). 2021 May 21;10(6):1033. doi: 10.3390/plants10061033 (PMC8224052; doi:10.3390/plants10061033)
Supplement: Supplementary file 1 [file plants-10-01033-s001.zip › Supplementary figures.pdf]

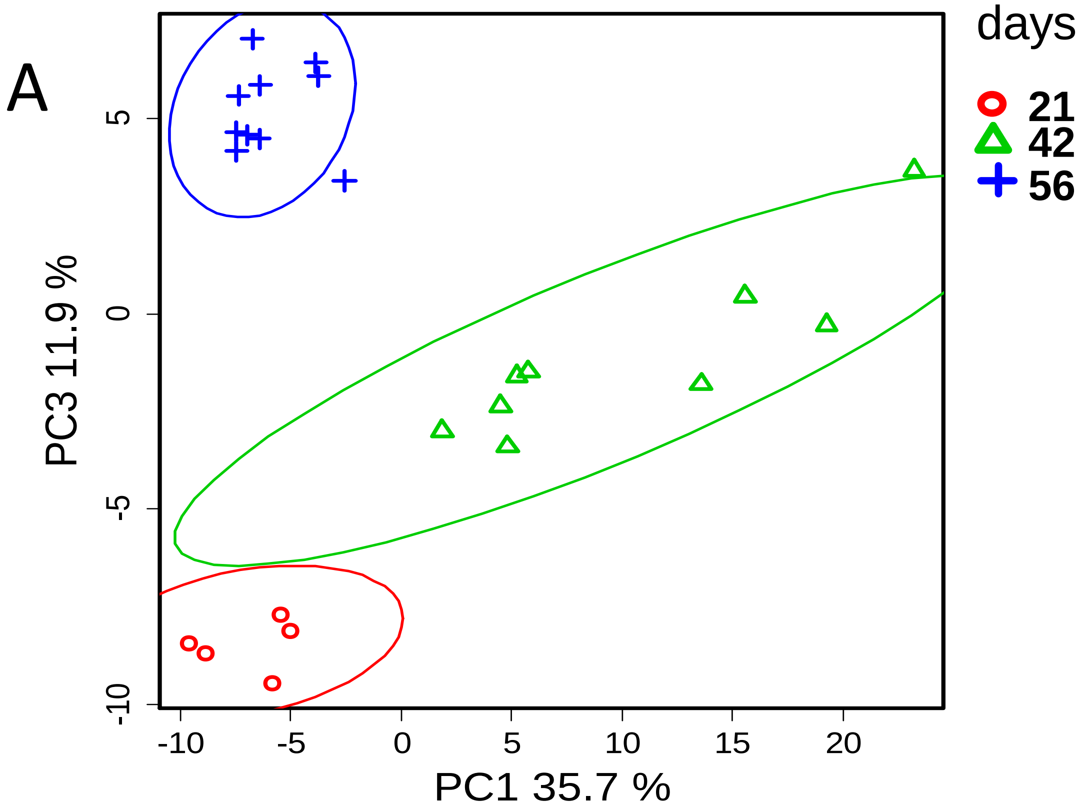

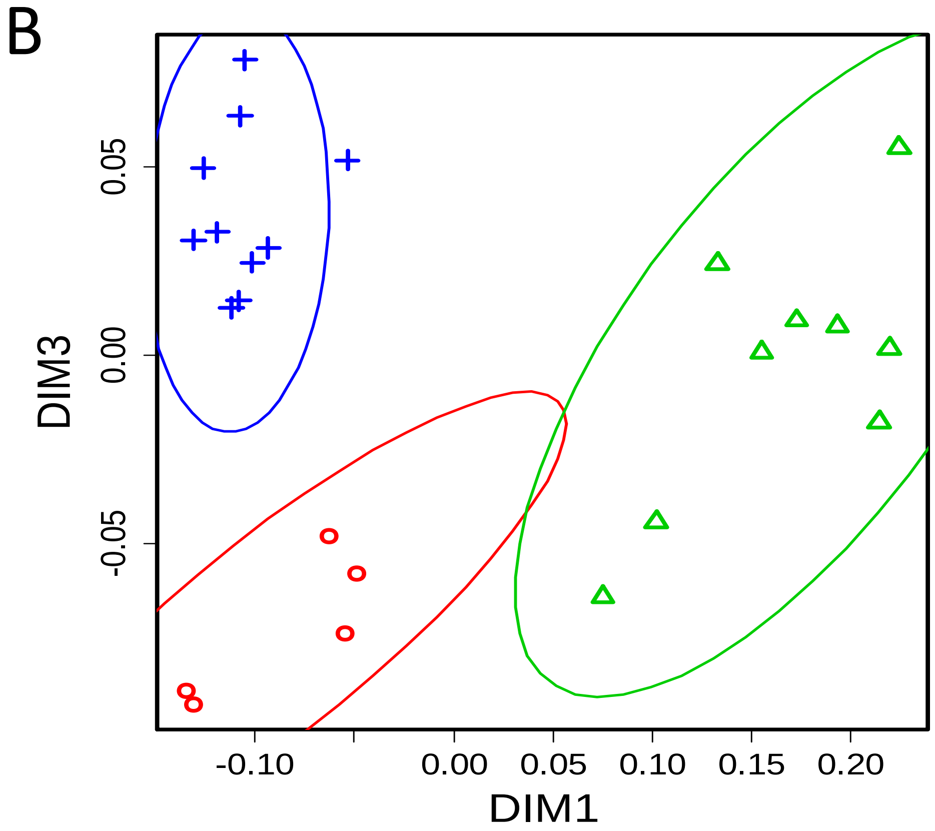

Figure. S1 Representation of metabolite profiles in lower dimensional space .

A. Principal component analysis (PCA) score plot, % - the proportion of variance associated with the principal component (PC), ellipses - 95% confidence intervals.

B. Representation of metabolite profiles in low-dimensional space obtained using the Multidemensional scaling (MDS) using 1-*ρ* as a distance measure, where *ρ* is Spearman's correlation coefficient.

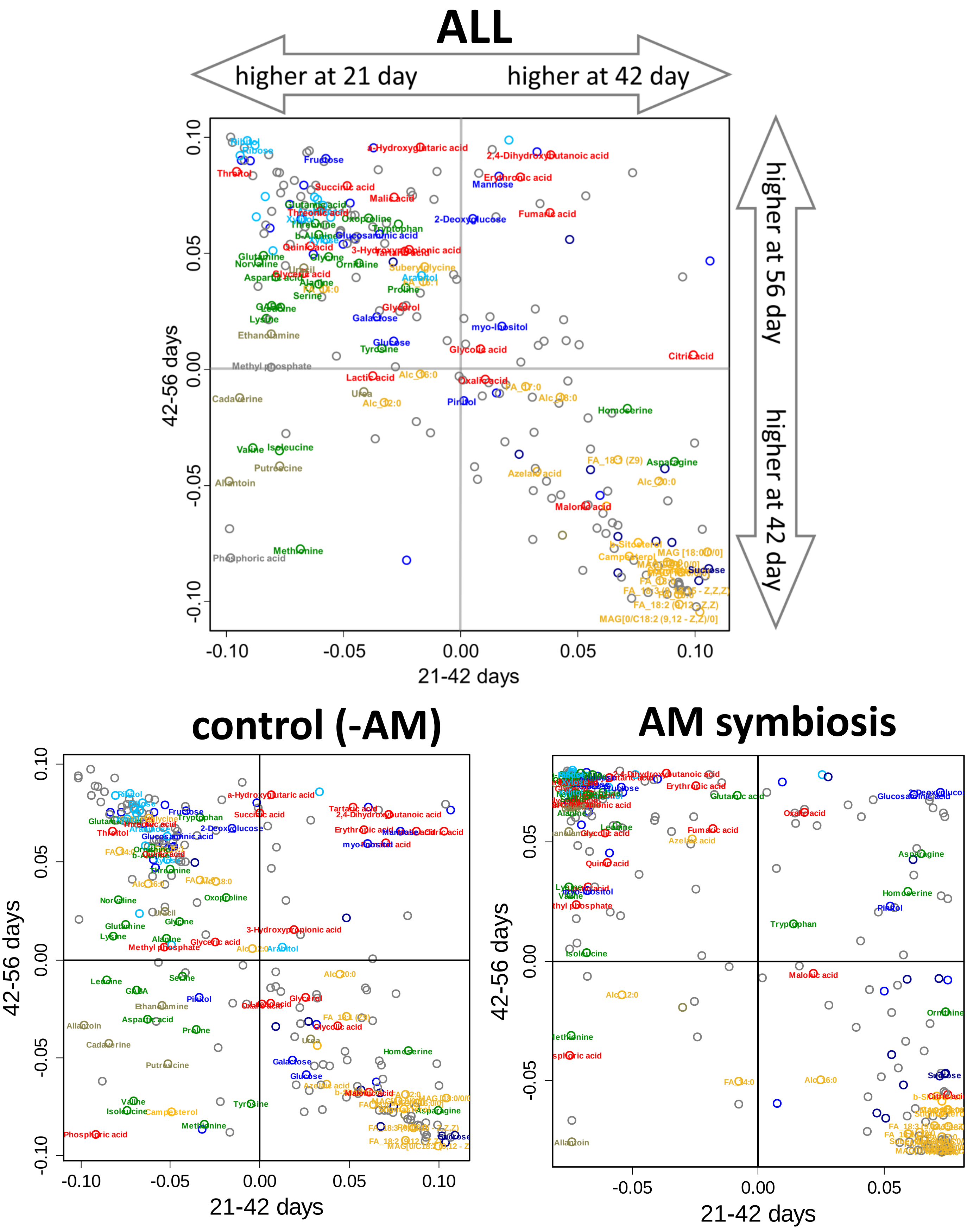

Figure. S2 SUS-plots: metabolites scattering in the space of loadings from OPLS-DA models for comparison roots of 21 and 42 days old plants (abscissa) and 42 and 56 days old (ordinate), positive values correspond to higher level at a later stage.

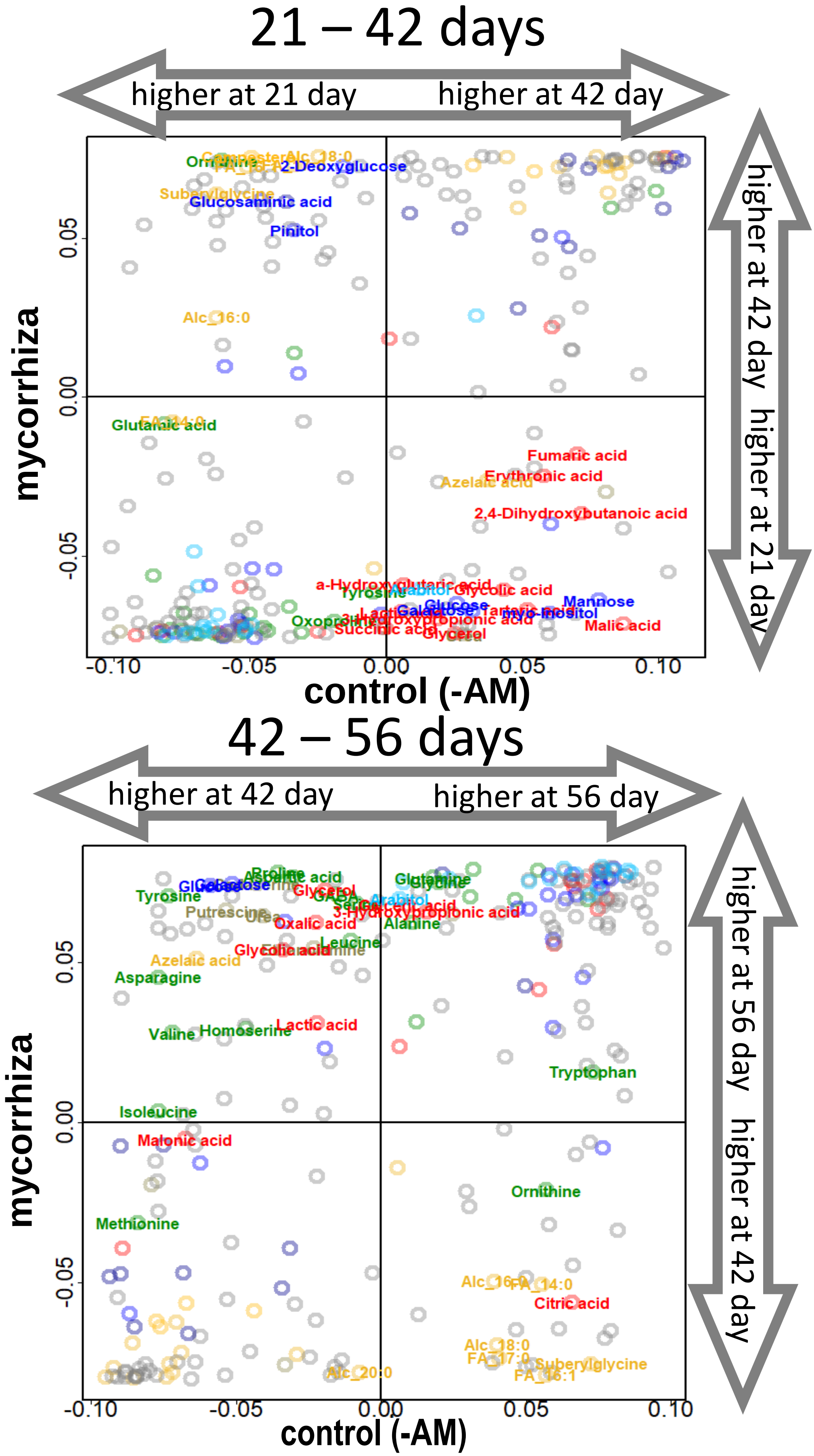

Figure S3. Influence of mycorrhiza on the metabolome dynamics. Metabolites scattering in the space of the loadings from OPLS-DA models for comparison adjacent timepoints under control (-AM, abscissa) and mycorrhization (ordinate). Metabolites which demonstrated strong difference in predictive factor loadings are labeled

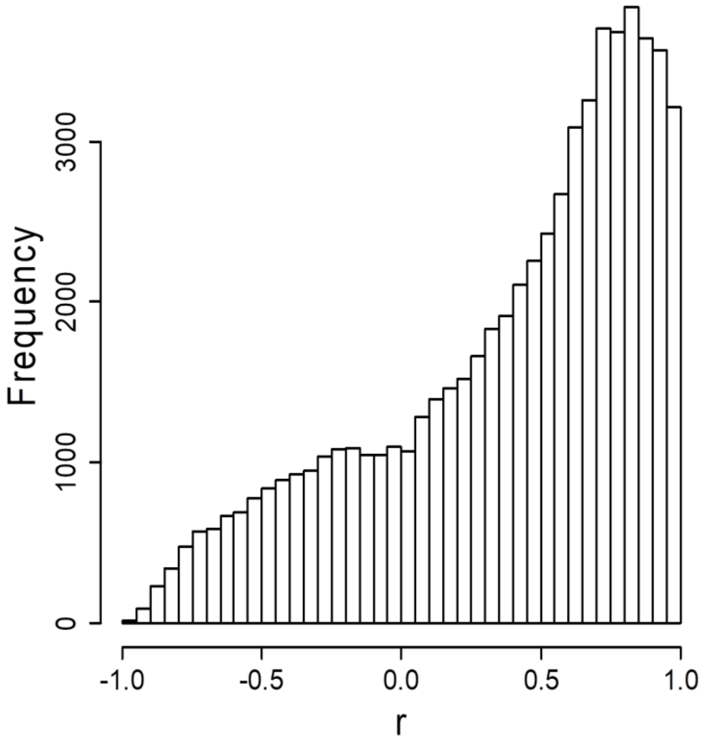

Figure S4. Histogram of Pearson's correlations (r) of metabolite mean normalized level in *Pisum sativa* roots sampled at 21, 42 an 56 days under mycorrhization or without.

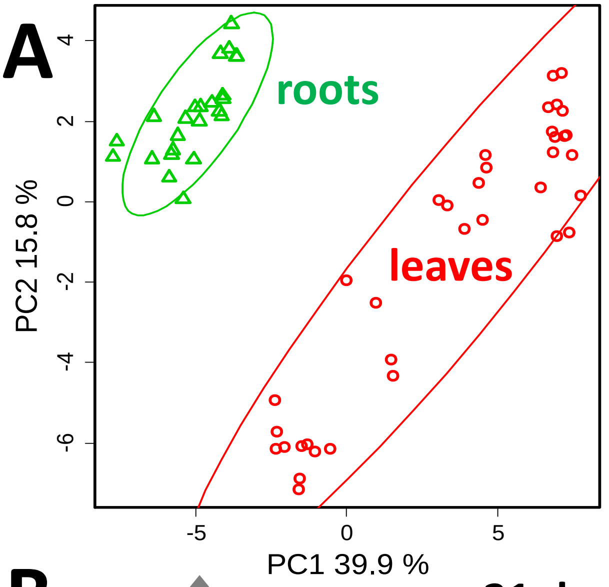

Metabolites were used in roots and leaves comparison.

3-Hydroxypropionate	FA_18:2 (9,12 - Z,Z)	Oxoproline	Malonic acid
Alanine	FA_18:3 (9,12,15 - Z,Z,Z)	pent_acid_RI=1761	Mannose
Arabinose	Fructose	pentose_RI=1697	Methyl phosphate
Arabitol	Fumaric acid	Phosphoric acid	myo-Inositol
Asparagine	GABA	Proline	Succinic acid
Aspartic acid	Galactose	Putrescine	Sucrose
b-Alanine	Glucose	Ribitol	Threitol
Campesterol	Glutamic acid	Ribose	Threonic acid
Citric acid	Glutamine	Serine	Threonine
disach_RI=2328	Glyceric acid	Stigmasterol	trisach_RI=3414
disach_RI=2866	Glycerol	Isoleucine	Tyrosine
Erythronic acid	Glycine	Lactic acid	Urea
Ethanolamine	hexonic_acid_RI=1824	Leucine	Valine
FA_16:0	hexose_RI=1865	Lysine	Xylitol
FA_17:0	hexose_RI=1887	MAG[0/16:0/0]	Xylose
FA_18:0	Homoserine	MAG[16:0/0/0]	
FA_18:1 (Z9)	Oxalic acid	Malic acid	

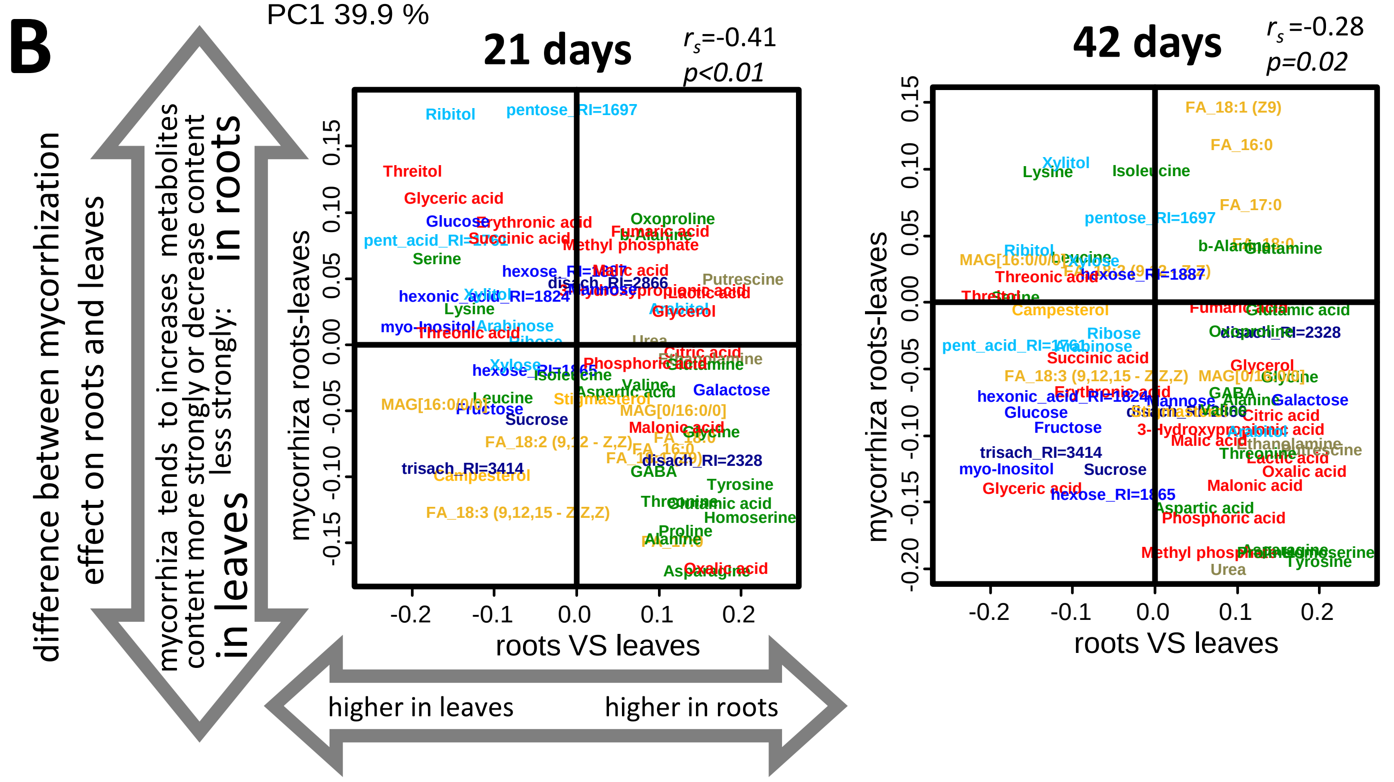

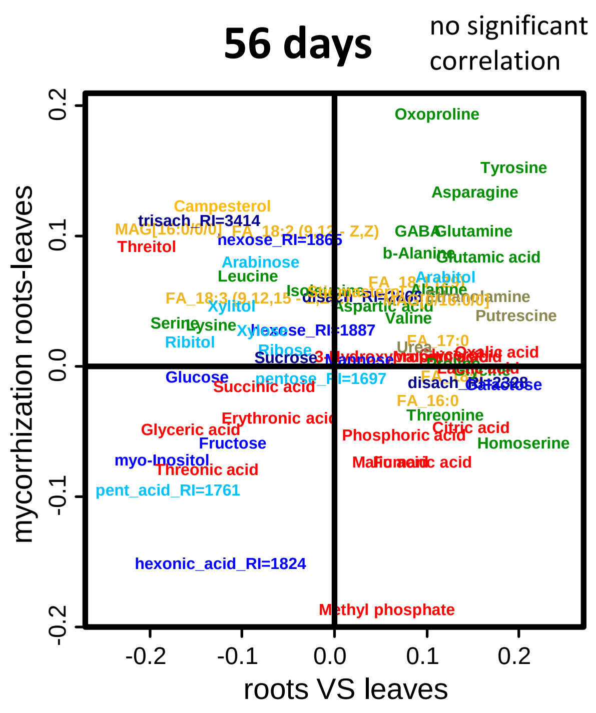

Figure S5. Metabolomic differences between leaves and roots and their relation to mycorrhiza effects difference.

A. PCA score plot, ellipses – 95% CI.

B. Scattering plots in the space of ordinate representing difference between loadings of predictive components (p_{roots}-p_{leaves}) from OPLS-DA models for control (-AM) and mycorrhized plants classification (positive loadings correspond to higher content under mycorrhization) and abscissa representing loadings of predictive components from OPLS-DA models for roots and leaves classification (positive loadings correspond to higher content in roots). r_s – Spearman's rank correlation.
